# Roadmap: Numerical-Experimental Investigation and Optimization of 3D-Printed Parts Using Response Surface Methodology

**DOI:** 10.3390/ma15207193

**Published:** 2022-10-15

**Authors:** Hamid Reza Vanaei, Sofiane Khelladi, Abbas Tcharkhtchi

**Affiliations:** 1 Léonard de Vinci Pôle Universitaire, Research Center, 92916 Paris La Défense, France; 2 Arts et Métiers Institute of Technology, CNAM, LIFSE, HESAM University, 75013 Paris La Défense, France; 3 Arts et Métiers Institute of Technology, CNRS, CNAM, PIMM, HESAM University, 75013 Paris La Défense, France

**Keywords:** FFF, RSM, temperature evolution, inter-layer bonding, mechanical strength

## Abstract

Several process variables can be taken into account to optimize the fused filament fabrication (FFF) process, a promising additive manufacturing technique. To take into account the most important variables, a numerical-experimental roadmap toward the optimization of the FFF process, by taking into account some physico-chemical and mechanical characteristics, has been proposed to implement the findings through the thermal behavior of materials. A response surface methodology (RSM) was used to consider the effect of liquefier temperature, platform temperature, and print speed. RSM gave a confidence domain with a high degree of crystallinity, Young’s modulus, maximum tensile stress, and elongation at break. Applying the corresponding data from the extracted zone of optimization to the previously developed code showed that the interaction of parameters plays a vital role in the rheological characteristics, such as temperature profile of filaments during deposition. Favorable adhesion could be achieved through the deposited layers in the FFF process. The obtained findings nurture motivations for working on the challenges and bring us one step closer to the optimization objectives in the FFF process to solve the industrial challenges.

## 1. Introduction

With the fast development of technology, the role of manufacturing techniques is more prominent, and industries are focusing more on finding faster techniques [1,2]. In the 19th century, the existence of several challenges in producing complex structures forced researchers to focus more on manufacturing techniques, which led to the appearance of new technology named rapid prototyping (RP) [3,4]. The additive manufacturing (AM) process encompasses innovative techniques facilitating the rapid construction of three-dimensional (3D) physical articles directly from computer-aided design (CAD) data [5,6].

Despite the large variety of AM techniques for the fabrication of thermoplastic polymers, metals, or ceramics [7,8], fused filament fabrication (FFF) is broadly utilized for prototype production in automotive, aerospace, optic, robotic, medical, and many more industries [9,10,11,12,13]. In this process, an extruder deposits a filament while moving in successive X-Y planes along the Z direction to construct a 3D part layer-by-layer [14]. Accordingly, the hot filament is deposited onto or beside filaments that were previously deposited, resulting in a temperature gradient between deposited filaments, and thus causing a cyclic temperature profile [15]. The mechanism of layer-by-layer deposition, as well as the thermal energy generated by the molten polymer, provoke the bonding of adjacent filaments [16]. Many researchers have mentioned that the strength and quality of 3D-printed constructs are affected by the extent of the bonding [17,18], which itself is dependent on the adhesion of deposited filaments [19].

FFF is extensively used to produce prototypes for applications, e.g., in the aerospace, medical, and automotive industries [20,21]. In this process, a polymer is fed into an extruder that extrudes a filament while moving in successive X-Y planes along the Z direction to fabricate a 3D part in a layer-by-layer process. Consequently, as the deposition takes place, the younger filament is deposited onto filaments that were previously deposited and are now in the cooling process. This causes their re-heating, defining a time when the interfaces of contacting filaments are above the glass transition temperature (Tg) in the case of amorphous material, or of the crystallization temperature (Tc) for semi-crystalline materials, which is necessary for proper bonding to take place. Therefore, each filament should be sufficiently hot during deposition, but not too hot, to avert deformation due to gravity and the weight of the filaments deposited in subsequent layers [22]. A thorough investigation of the literature reveals the following limitations. (1) An overview performed on the influence of process parameters through the part quality fabricated by the FFF process appears to conflict with their results. For example, a study in 2002 concluded that layer thickness has a less significant influence on the tensile strength. After three years, other researchers found that the tensile strength of an FFF part first decreased and then increased as layer thickness increased. A few years later, in 2010, another study proposed that layer thickness has a low impact on the tensile strength. These consequences call for a comprehensive investigation through the FFF parameters. (2) FFF parameters not only affect the part quality, but also greatly influence the build time involved. However, studies found in the literature did not focus on the influence of process parameters on the build time. (3) Almost all research has focused on investigating one material at a time, or even one parameter at a time. In contrast, there are a number of parameters in reality that play an essential role during production. Furthermore, based on the various research studies, it is required to investigate the simultaneous effect of important parameters to better understand the FFF parameters. (4) A thorough Investigation of the combined effects of the FFF parameters is required, which helps understand each parameter’s influence further, with their interaction on the bond quality. This point of view helps optimize the FFF process to reach the final goal of improving bond quality [23,24,25,26,27].

Accordingly, several parameters affect the manufactured part quality, such as the temperature profile of the polymer, and thus the inter-layer bonding. It is therefore important to understand how the process parameters affect the evolution of the filament’s temperature, as mentioned. The optimization could be obtained by the maximization of mechanical characteristics and bonding quality (objective: part quality), and by the minimization of part cost and build time (objective: process optimization). Diffusion and neck-growth between two adjacent filaments would be affected by changes in the environment or platform temperatures, confirming the significance of heat transfer in this process. Regarding the applied material and studied parameters, it was found that almost all researchers tried to consider the influence of parameters by different methods of characterization (e.g., tensile or bending) by using a unique parameter at different values [23,27,28,29,30,31,32,33,34,35,36,37].

To evaluate the quality of the final part by considering the effect of several variables, many researchers have applied the Taguchi method [30,38,39]. Using the Taguchi method, the impact of these variables on the mechanical properties, surface roughness, and dimensional accuracy have been thoroughly investigated [40,41]. To perform this action, the response surface methodology (RSM) is categorized as a promising technique for optimization purposes by combining numerous independent factors to achieve an interaction between the desired variables. In fact, the mechanism of implementing RSM is based on the design of experiment (DOE) approaches (e.g., least square fit, central composite design, etc.) to apply the findings through the verification steps by the analysis of variance (ANOVA) [42]. Several works on optimization purposes use the Taguchi method or RSM to investigate the influence of individual parameters on the mechanical strength or dimensional accuracy of 3D-printed parts. Almost none of them performed a thorough investigation by taking into consideration the interaction of individual parameters.

Given the above-mentioned statements, the temperature evolution during the FFF process thoroughly specified the fabricated structures’ quality and mechanical strength. Experimental monitoring and analytical investigations are, however, challenging in FFF; a lack of useful knowledge relates to the problem of bonding in this process. Since the rheological characteristics, such as thermal properties, are a function of temperature, the mentioned process variables are widely affected by the temperature evolution of filaments while printing. To sum up, the investigation on optimizing FFF materials while printing is still in its early stage and governs the bonding quality itself.

The present work aims to cover the still challenging points by focusing on the most critical variables, such as the temperature profile of filaments during deposition. In fact, statistical DOE techniques have been used to express the influence of process variables on the physico-chemical, mechanical, and thermal characteristics of the 3D-printed parts to isolate the optimized conditions, according to a response surface plot. The optimized zone was then used as an input to our numerical approach that has previously been developed and validated for predicting the temperature profile of deposited layers within the optimized zone. This paper is structured as follows: Section 2 presents the material and the experimental characterizations; Section 3 expresses a summary of the numerical approach used in this paper; Section 4 explains the obtained results using RSM and its findings through the previously developed numerical code; and the overall conclusion is presented in Section 5.

## 2. Materials and Methods

### 2.1. Material, 3D Printer, and Sample Preparation

**Figure 1:**
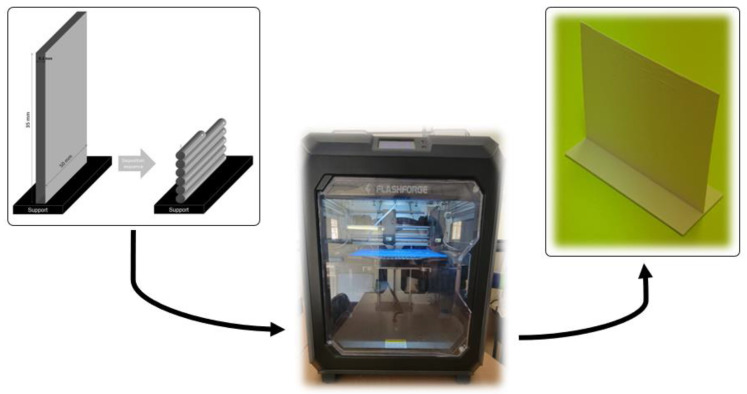
Representation of the solid bock (vertical wall), applied FFF machine, and the printed solid block.

A commercial PLA filament (purchased from Fillamentum) was applied in this study. In order to print the solid blocks, a ‘Flashforge Creator 3’ was then utilized as a FFF machine for printing the solid blocks. The characteristics of the filament include a diameter of 1.75 (±0.01) mm, a density of 1.24 g.cm^−3^, and a melting temperature of ~150 °C. A unidirectional motion of the extruder was considered for constructing a vertical wall, including the deposition of filaments on top of each other (See Figure 1).

A desktop 3D printer was then used to fabricate the solid blocks (vertical wall), considering the process parameters of the employed machine. All printing parameters used in this work are summarized as shown in Table 1.

Presumably, three samples per parameter set were utilized and the sampling position for the characterization techniques have been implemented in the same way as the previous work of authors [43]. It is worth mentioning that the process variables are fixed based on the previous experiences and research works of the authors [44].

### 2.2. Characterization Methods

#### 2.2.1. Differential Scanning Calorimetry (DSC) Analysis

To study the thermal properties of PLA, DSC was applied using a TA instrument Q1000 system. Samples were heated from ambient temperature to 200 °C with a heating rate of 10 °C.min^−1^ under 50 mL.min^−1^ of nitrogen flow. The sample weight varied in the range of 6–10 mg. Using the TA universal analysis software, the enthalpies at different temperatures from both exothermic and endothermic peaks were determined. Additionally, the degree of crystallization ($X_{c}$) was calculated using the following equation:

(1) $$X_{c}=\left(\Delta H_{m}-\Delta H_{c}\right)/\Delta H_{m}^{0}$$

where ΔH_c_ and ΔH_m_ are cold crystallization and melting enthalpies, respectively, and the melting heat ($\Delta H_{m}^{0}$) of 100% crystalline PLA is considered equal to 93.7 j.g^−1^, according to the literature [45].

#### 2.2.2. Mechanical Testing (Quasi-Static Tensile Test)

A tensile test until failure is implemented using an INSTRON4301 machine. The specimen geometry used to cut samples from the printed vertical wall is based on the ISO 37-3. In fact, a mold with the desired geometry, according to the mentioned standard, has been used for cutting the samples from the 3D printed vertical walls. The loading velocity is fixed at 1 mm.min^−1^.

### 2.3. Design of Experiments (DOE)

In order not to perform too many tests, DOE is a promising technique for the consideration of the influence of engaged parameters on a specific characteristic. Accordingly, central composite design (CCD) is considered as a DOE method in this study. The objective is to determine the number of experiments that are required for optimization purposes. To achieve this, liquefier temperature (T_L_), platform temperature (T_P_), and print speed (V_L_) have been considered as the process variables. For each parameter, four responses as the degree of crystallinity ($X_{c}$), Young’s modulus (E), tensile strength (σ_max_), and elongation at break (ɛ) have been taken into account for optimization purposes.

To find the relation through the control variables (T_L_, T_P_, and V_L_) and response variables ($X_{c}$, E, σ_max_, and ɛ), we consider using the response surface methodology (RSM) by taking into account the CCD. The aim is to formulate the response as a function of controlled experimental variables and to obtain the best set of them that provide the best response values. Using a second-order polynomial RSM, the recorded experimental data can be fitted by Equation (2) [46]:

(2) $$Y=\beta_{0}+\overset{N}{\underset{i=1}{\sum }}\beta_{i}\;X_{i}+\overset{N}{\underset{i=1}{\sum }}\beta_{ii}\;X_{i}^{2}+\;\overset{N}{\underset{i\neq j}{\sum }}\beta_{ij}\;X_{i}X_{j}+\;\varepsilon \;$$

where *β* is the mode constant, $\beta_{i}$ is the linear coefficient, $\beta_{ii}$ is the quadratic coefficient, $\beta_{ij}$ is the cross-product coefficient, ε is the experimental error term, and *Y* is the predicted response. Furthermore, $X_{i}$ and $X_{j}$ (*i* < *j*) act as those variables that have been defined for each experimental run.

Based on our previous works, we have considered a range for the studied variables: T_L_ ∈ [200–230 °C], T_P_ ∈ [50–100 °C], and V_L_ ∈ [20–60 mm.s^−1^]. Then, an analysis of variance (ANOVA) was implemented to consider the impact of process variables on response parameters. Based on the obtained results of the studies obtained by El Magri et al. in the optimization of printing parameters on 3D-printed PEEK [39] and PPS [46] parts, a one-way ANOVA was applied to estimate the statistical parameters. The objective was to monitor the significant differences between the average value of the process variables by defining the probability (*p*-value) and the determination coefficient of the model (R^2^). To achieve this, Minitab 18.0^®^ (PA, USA) was applied to set up the DOE further with the statistical model to plot the response surface for the optimization of the process variables.

## 3. Temperature Evolution and Heat Transfer

**Figure 2:**
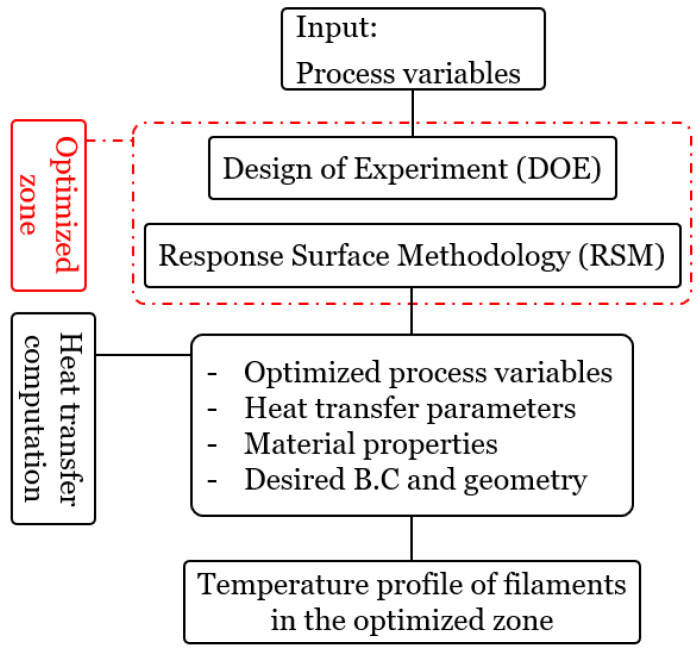
General flowchart of the proposed approach in this study.

The quality and mechanical strength of the 3D-printed parts are extensively affected by the temperature evolution of filaments during layer deposition [47]. As this evolution and cooling of filaments is a transient process, several parameters actively change this evolution [48,49]. Despite the several works that have been accomplished through evaluating the mentioned fact, researchers are still trying to perform optimization approaches in this regard. To extend the knowledge of this issue, heat transfer, and consequently the temperature evolution of filaments, should be thoroughly investigated. The heat transfer model previously developed by authors has been applied here to know how to consider optimization purposes by including the heat transfer mechanisms [50]. The developed C++ computer code is based on the finite volume method (FVM), performing a platform to evaluate and predict the heat transfer mechanism during the gradual deposition of filaments. A flowchart of the utilized approach further with the previously developed code is shown in Figure 2.

As described in the previous work, a heat transfer balance over a given infinitesimal volume (a vertical wall in this case) has been implemented to determine the volume integrals of a partial differential equation to the full surface.

## 4. Results and Discussions

### 4.1. DSC and Tensile Analysis

In previous studies, it has been widely reported that the process variables, such as liquefier temperature, platform temperature, and print speed, could play an important role in the characteristics of the 3D-printed parts [23,24,26,30]. In fact, the mentioned parameters have a significant impact on the fluidity and also the solidification of the extruded filaments. Accordingly, prior to DSC analysis, samples were printed based on the conditions presented in the previous section. The presented results in Table 2 show that an increase in the liquefier temperature at the fixed values of platform temperature and print speed resulted in higher degree of crystallinity. Although there is a periodical increase and decrease with the variation of platform temperature and print speed, the increase of the mentioned parameters also tends to increase the degree of crystallinity. This means that controlling the parameters related to the temperature variation and solidification of the layers can control the degree of crystallization and inter-penetration of materials. One can note that the higher the crystallization, the higher the bonding between the crystallized parts.

Furthermore, it has been widely indicated that the mentioned process variables play a crucial role in the mechanical strength and bonding between the deposited layers toward the applicable materials in the FFF process [35,51,52,53,54]. Hence, as previously mentioned, we have considered printing the samples in different conditions. The objective is to consider the mechanical characteristics by determining the tensile strength, Young’s modulus, and the elongation at break. According to the presented results in Table 2, the ultimate strength increases as much as the liquefier temperature increases. However, considering other conditions with the variation of the platform temperature or print speed, there is also an increase in the mentioned features. Young’s modulus is another indicator of comparing the strength of a material, and the same observation was concluded by comparing the obtained values. The elongation at break could be a criterion for considering the ductility of the 3D-printed material. In fact, by comparing the recorded values, it was observed that the more we keep the filaments hot by reducing the cooling rate, the more we would have ductility. This could be related to the fact that the crystallization can be slow or rapid for polyesters. For PLA, the crystallization depends on the material’s crystallization rate and the cooling rate. The more it has time for crystallization, the more inter-penetration of the material could have resulted between layers, and better bonding could be obtained.

### 4.2. RSM Fitting

Using RSM, liquefier temperature (T_L_), platform temperature (T_P_), and print speed (V_L_) have been categorized as the factors further with the degree of crystallinity ($X_{c}$), Young’s modulus (E), tensile strength (σ_max_), and elongation at break (ɛ) as the measured responses. In fact, the idea is to take into account the regression model of the mentioned responses from RSM as a function of the factors (process variables) using the following equation:

(3) $$y_{i}=f_{p}\;\left(T_{L_{i}^{p}}.\;T_{P_{i}^{p}}.\;V_{L_{i}^{p}}\right)+\varepsilon_{i}^{p}\;$$

where $y_{i}$ is the response, *p* is the observation number, and ε is the residual.

**Figure 3:**
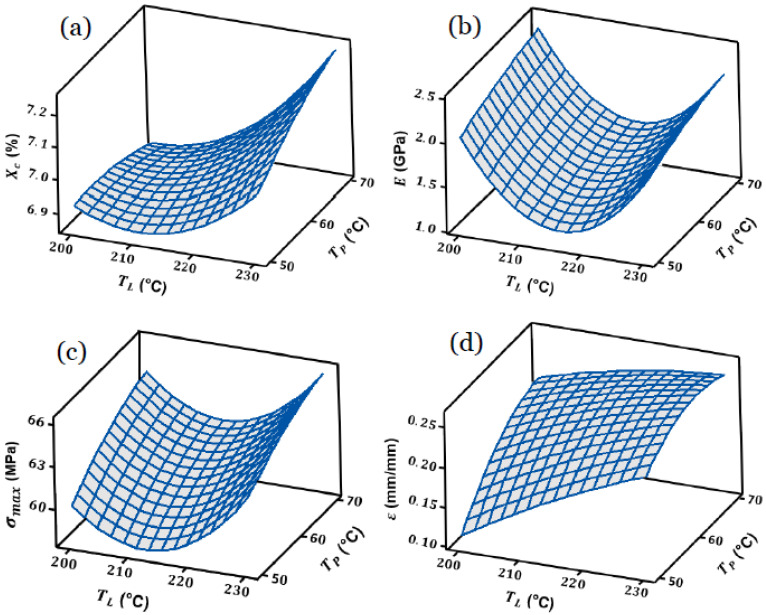
Response surfaces: (**a**) degree of crystallinity, (**b**) Young’s modulus, (**c**) tensile strength, and (**d**) elongation at break as a function of the process variables.

Here, the objective is to take advantage by validating the performance of the obtained models using ANOVA analysis. It will facilitate analyzing the obtained results of the established models and calculating the desired coefficients of the regression model for each response, as presented previously. According to the mechanism of RSM, the evaluation of the optimum state according to the defined factors maximizing the responses, it is possible to obtain the response surfaces of the models based on the defined factors, liquefier temperature (T_L_), platform temperature (T_P_), and print speed (V_L_), respectively. Figure 3 indicates the response surface of the degree of crystallinity ($X_{c}$), Young’s modulus (E), tensile strength (σ_max_), and elongation at break (ɛ) as a function of the process variables (factors). It includes the ideal conditions by which the response variables perform the best quality of the final 3D-printed parts. To perform this step, a multi-response optimization technique could be applied and plot the results in a 3D graphical curve, including each response as a function of the defined factors. By holding the print speed (V_L_), Figure 3a–d includes the response surfaces as a function of liquefier temperature (T_L_) and platform temperature (T_P_) for the maximum degree of crystallinity, Young’s modulus, tensile strength, and elongation at break, respectively.

The mentioned features result in the optimal area (optimization zone according to the process variables of the FFF process) in which the degree of crystallinity, Young’s modulus, tensile strength, and elongation at break are at their maximum value. The optimized region according to the obtained responses has been presented in the next section, representing the area that has the following criteria: $X_{c}$ > 7%, E > 2.1 GPa, σ_max_ > 65 MPa, and ε > 0.24%.

### 4.3. Response Optimization

The input variables used in RSM are the main criteria in this evaluation technique. By controlling these variables, it is possible to optimize the obtained responses for process optimization.

**Figure 4:**
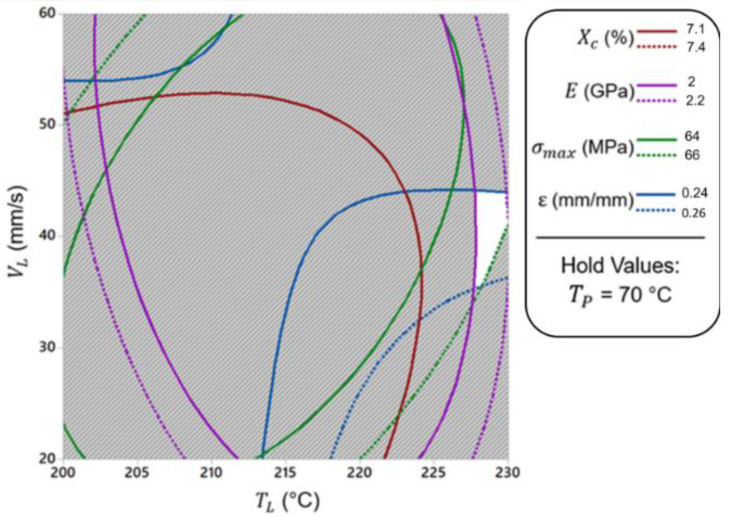
Multi-response optimization with reference to the liquefier temperature and print speed.

Presumably, one useful output of RSM is the optimization of the responses [55]. Here, we have performed a multi-response optimization technique to carry out an interaction through the variables or process parameters. To achieve this, the best combination of those responses has been taken into account, resulting in maximization. Using Minitab 18.0^®^, an overlaid contour plot was utilized, letting us recognize the acceptable range through all responses by plotting them together with the respective limit. As shown in Figure 4, the white zone corresponds to the desired area, which comprises all responses. In other words, recognizing this zone leads to the minimization of the variation of the engaged parameters, and thus the optimized zone corresponding to the interaction of the variables could be determined.

In this case, the platform temperature was held at T_P_ = 70 °C, as there was the maximum level of responses at this value. Considering these sets of contours, the limitation of the fitted responses could be obtained for the acceptable values. The contour for each response has been shown in different colors representing the solid line for the lower limit and the dotted line for the upper limit, respectively. Therefore, the obtained criteria for each response, including lower and upper limit, have been applied to the graphical optimization. The white zone expresses the zone by which all criteria are satisfying. As can be seen from Figure 4, the accepted liquefier temperature was observed to be 227 < T_L_ < 230 °C, and the print speed was observed to be 35 < V_L_ < 45 mm.s^−1^ with the platform temperature T_P_ = 70 °C.

In this regard, some optimized conditions have been extracted from the previous findings to implement them through the developed code to recognize the temperature evolution according to the optimized parameters (see Table 3). Apparently, the object is to implement the characteristics of the fabricated parts according to the physico-chemical and mechanical properties further with the process variables.

### 4.4. Optimized Temperature Profile of Filaments

The heat transfer model developed in our previous work has been implemented here for optimization purposes [50]. The main feature of the previously developed model is as follows: it is general and applicable to different types of materials, whether amorphous/semi-crystalline or polymer/composite. It is also possible to include all engaged parameters and process variables for temperature and heat transfer evaluations.

It is broadly believed that controlling the cooling of filaments during deposition is an important issue for optimization purposes. In our case, for PLA, the temperature profile of filaments during the cyclic cooling and re-heating should have remained above the crystallization temperature (T_C_) for proper bonding [56]. The higher the temperature of filaments, the better the crystallization, and thus the adhesion of the deposited layers [57].

Given the above-mentioned explanations and according to the defined flowchart (see Figure 2), we have considered applying the present findings to the temperature variation of filaments to compare with our previously discussed results. To achieve this, we have deemed the three extracted conditions according to Table 3 with the specified condition of the previous work for four random points as follows:

- **Reference Condition**: T_L_ = 210 °C, T_P_ = 50 °C, V_L_ = 20 mm.s^−1^
- **Condition No. 1**: T_L_ = 230 °C, T_P_ = 70 °C, V_L_ = 35 mm.s^−1^
- **Condition No. 2**: T_L_ = 230 °C, T_P_ = 70 °C, V_L_ = 40 mm.s^−1^
- **Condition No. 3**: T_L_ = 230 °C, T_P_ = 70 °C, V_L_ = 45 mm.s^−1^.

**Figure 5:**
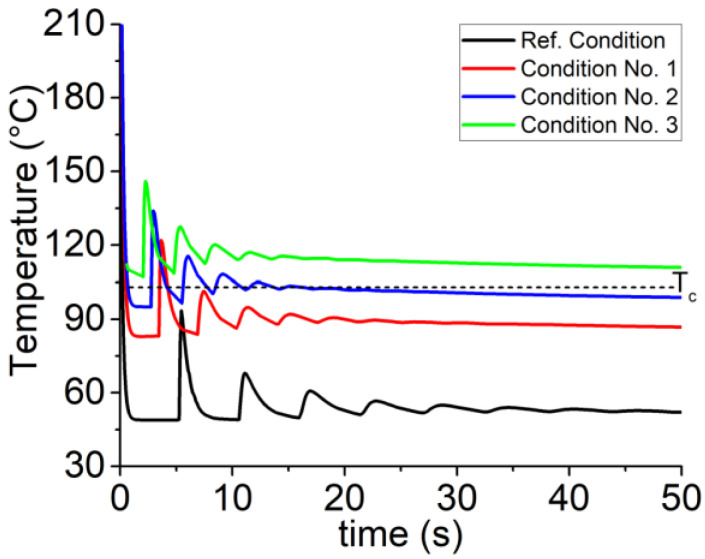
Temperature evolution during the deposition of the first layer of a vertical wall, according to the defined conditions.

Figure 5 reveals significant differences in the temperature profile of different layers for the implemented conditions. As can be seen, there is a significant shift and increase between the reference condition and the optimized conditions obtained from RSM. The following statements are expected by taking into account the individual effect of each parameter:

- **Liquefier temperature**: Negligible effect on the cooling rate and onset of the re-heating peaks.
- **Platform temperature**: Considerable effect on the entire temperature profile and onset of the re-heating peaks.
- **Print speed**: Considerable effect on the occurrence and onset of the re-heating peaks.

However, a comparison through the reference condition and condition No. 1 represents that, although there is a great shift to the onset of peaks by increasing print speed, and thus reducing the deposition time, they all have identical onsets. Obviously, the increase of platform temperature tends to decrease the cooling rate, and thus there exists a great shift through the temperature profile of the filaments. Apparently, the temperature profile of filament varies and increases up to the crystallization temperature (T_c_) and helps the semi-crystallized materials (in our case: PLA) to have better bonding due to better crystallization and inter-penetration of material between the adjacent layers.

Accordingly, by taking into account the conditions No. 1–3 with a difference in the print speed, the temperature profile of filaments remains high enough during the deposition. The most important feature is the difference between conditions No. 1 and No. 3 while increasing the print speed from V_L_ = 35 mm.s^−1^ to V_L_ = 45 mm.s^−1^, causing ~25 °C shift through the entire temperature profile of the filament. As shown in Figure 5, the related curve is above the crystallization temperature. It is believed that better crystallization and adhesion of the layers occur if the filaments’ temperature profile remains in this zone (T_c_ < T_desired_ < T_m_).

## 5. Conclusions

This study presents a numerical-experimental roadmap toward optimizing the FFF process by taking into account some physico-chemical and mechanical characteristics to be implemented in the thermal behavior of the material using a response surface methodology (RSM). It aimed at exploring the optimized zone by analyzing the individual effects of the liquefier temperature, platform temperature, and print speed. At the early stage, RSM gives a confidence domain with a high degree of crystallinity, Young’s modulus, maximum tensile stress, and elongation at break. The extracted zone of optimization expressing an interaction of parameters was then incorporated into the previously developed code by the authors. Compared with the previous work, the main characteristic includes the interaction of the process variables, resulting in a better quality of the final part.

The predicted temperature profile of filaments showed that the interaction of parameters plays an important role in the rheological characteristics, such as the temperature profile of filaments during deposition. It is believed that by controlling the temperature variation, favorable adhesion could be achieved through the deposited layers in the FFF process. The obtained findings nurture motivations for working on the challenges and bring us one step closer to the optimization objectives in the FFF process to solve the industrial challenges.

## Figures and Tables

**Table 1:** Process variables of the 3D-printing process.

Parameter	Value
Liquefier temperature T_L_ (°C)	200–230
Platform temperature T_P_ (°C)	50–100
Print speed V_L_ (mm.s^−1^)	20–60
Layer height (mm)	0.2
Infill (%)	100
Filament cross-section	Circular

**Table 2:** Various characteristics for PLA samples at various runs.

Run Order	Factors			Responses			
	T_L_ (°C)	T_P_ (°C)	V_L_ (mm.s^−1^)	$X_{c}$	E (GPa)	σ_max_ (MPa)	ɛ (mm.mm^−1^)
1	200	50	40	6.95	2	60	0.2
2	210	70	40	6.9	1.2	58	0.21
3	210	50	60	7.25	1	56.5	0.23
4	210	50	40	6.83	1.1	56.5	0.18
5	220	50	60	7.1	1.8	64	0.25
6	230	70	60	7.1	2.1	66.5	0.23
7	220	50	20	7.25	1.4	62	0.12
8	200	70	20	6.8	1.9	59	0.194
9	220	50	40	7.1	1.5	62	0.21
10	230	50	20	6.83	1.1	57	0.105
11	230	70	20	6.8	1.4	59.5	0.27
12	220	70	40	7	1.5	62.5	0.25
13	200	70	40	6.8	2.1	62	0.19
14	200	50	20	6.72	1.8	59	0.1
15	230	50	40	7	2	65	0.26
16	210	50	20	5.12	1.2	60	0.097
17	220	70	60	7.1	1.9	65	0.26
18	210	70	20	6.83	1.3	61.5	0.25
19	230	70	40	6.9	2.2	66	0.27
20	200	50	60	6.87	2.1	62.5	0.18
21	220	70	20	7.5	1.5	63	0.23
22	200	70	60	6.9	2.1	63	0.2
23	230	50	60	6.95	2	64.5	0.27
24	210	70	60	7.1	1.2	57	0.25

**Table 3:** Optimized conditions according to the RSM fitting and optimization of the responses.

Condition No.	T_L_ (°C)	T_P_ (°C)	V_L_ (mm.s^−1^)
1	230	70	35
2	230	70	40
3	230	70	45

## Data Availability

Not applicable.
